# Correlation between intrasellar pressure, pituitary adenoma invasiveness, pituitary dysfunction, and apoplexy

**DOI:** 10.3389/fendo.2025.1700711

**Published:** 2025-12-17

**Authors:** Shan Xie, Zhilin Shao, Dongqi Shao, Xialin Zheng, Huadong Tang, Yu Li, Renhao Zhang, Tianyang Wu, Hao Lang, Rui Xu, Chenxi Li, Hongwei Cheng, Zhiquan Jiang

**Affiliations:** 1 Department of Neurosurgery, First Affiliated Hospital of Anhui Medical Univeisity, Hefei, China; 2 Department of Neurosurgery, First Affiliated Hospital of Bengbu Medical University, Bengbu, China; 3 Department of Emercysurgery, First Affiliated Hospital of Bengbu Medical University, Bengbu, China

**Keywords:** intrasellar pressure, pituitary adenoma, invasiveness, hypopituitarism, apoplexy

## Abstract

**Background:**

Few studies have reported the association between intrasellar pressure (ISP) and tumor invasiveness, hypopituitarism, or pituitary apoplexy in patients with pituitary adenomas. This study aimed to investigate the relationship between intraoperatively measured ISP and pituitary adenoma invasiveness, as well as to assess whether elevated ISP is associated with hypopituitarism and pituitary apoplexy.

**Methods:**

We retrospectively analyzed 84 patients with newly diagnosed pituitary adenomas who underwent endoscopic transsphenoidal surgery at the First Affiliated Hospital of Bengbu Medical University between January 2024 and March 2025. ISP was measured intraoperatively. Tumor invasiveness was assessed using the Hardy-Wilson and Knosp grading systems on preoperative MRI. Tumor volume was calculated with 3D-Slicer software. Spearman’s correlation analysis was used to evaluate associations between ISP, tumor volume, invasiveness, hypopituitarism, and pituitary apoplexy.

**Results:**

The mean intraoperative ISP was 30.91 ± 7.03 mmHg. Patients with Hardy-Wilson grade III–IV or Knosp grade 3–4 tumors had significantly higher ISP than those with lower grades. Tumor volume correlated positively with ISP, with tumor height showing the strongest correlation. Elevated ISP leads to a higher preoperative incidence of adrenal insufficiency, an increased risk of preoperative hypothyroidism, and a greater likelihood of preoperative hyperprolactinemia in patients with pituitary adenomas, but it shows no clear association with pituitary apoplexy. No significant correlation was observed between ISP and pituitary apoplexy or postoperative hypopituitarism at 12 weeks.

**Conclusion:**

ISP is strongly associated with tumor invasiveness and tumor volume in pituitary adenomas. Elevated ISP increases the risk of preoperative adrenal insufficiency, hypothyroidism, and hyperprolactinemia, but does not appear to affect pituitary apoplexy or postoperative hypopituitarism at 12 weeks.

## Introduction

Pituitary adenomas represent the most common neoplasms of the sellar region, comprising both functional tumors that autonomously secrete pituitary hormones and non-functional tumors without hormone hypersecretion (1). These monoclonal tumors are categorized as macroadenomas (maximum diameter ≥ 1 cm) or microadenomas (maximum diameter < 1 cm) (2). Clinically, approximately 1 in 1,100 individuals may develop significant symptoms attributable to pituitary adenomas. Progressive tumor enlargement can induce mass effects, leading to headache, hormone hypersecretion, visual field defects, and hypopituitarism (3). Compared with the general population, patients with hypopituitarism secondary to pituitary adenomas exhibit nearly a twofold increase in all-cause mortality (4). Thus, investigating pituitary dysfunction in this population holds important clinical significance.

Although most pituitary adenomas are benign and can be effectively managed with surgery and/or pharmacotherapy, a subset displays invasive growth patterns. Invasive pituitary adenomas are often resistant to conventional therapeutic approaches—including surgery, medication, and radiotherapy—and are prone to early recurrence or regrowth after resection. Such behavior is considered a hallmark of poor prognosis (5). Intraoperative observations suggest that invasive pituitary adenomas constitute approximately 40% of all cases (6). These tumors frequently extend into the suprasellar, infrasellar, and cavernous sinus regions, and their invasiveness is typically classified on MRI according to the Hardy-Wilson and Knosp grading systems (7, 8).

The vascular supply of pituitary adenomas arises primarily from the superior and inferior hypophyseal arteries, the latter originating from the internal carotid artery (9). Owing to their unique anatomical location, pituitary adenomas often experience compromised blood supply. The sella turcica, a relatively rigid bony structure, normally shields the pituitary gland from trauma and fluctuations in intrasellar pressure (ISP). While ISP has never been measured in healthy pituitary glands, it is presumed to approximate normal intracranial pressure (ICP) (10). Prior studies indicate that tumor enlargement elevates ISP. Expanding adenomas may compress the inferior hypophyseal arteries; given the vascular constraints imposed by surrounding anatomy, this diminished blood supply contributes to further ISP elevation (11).

Rising ISP can compress the pituitary stalk, impairing normal hormone secretion and manifesting clinically as adrenal insufficiency, hypothyroidism, and hyperprolactinemia (12). However, findings from earlier studies regarding the relationship between ISP and pituitary adenoma growth patterns have been inconsistent. To date, no definitive evidence has established a correlation between ISP and tumor invasiveness or tumor volume, and few studies have clarified whether ISP contributes to endocrine dysfunction in these patients. Pituitary apoplexy is a rare syndrome caused by ischemia or hemorrhage of the pituitary gland due to tumoral or non-tumoral factors, with clinical manifestations ranging from asymptomatic subclinical events to life-threatening conditions. Although current studies have identified pregnancy as a known risk factor for pituitary apoplexy, Kajal S. et al. suggested that, in most patients, tumor size (macroadenoma) and the non-functioning status of the adenoma are the only significant independent triggers of pituitary apoplexy. Nevertheless, its risk factors and underlying molecular mechanisms remain to be fully elucidated (13). Notably, no studies have explored the potential association between ISP and pituitary apoplexy.

Accordingly, this study was designed to evaluate intraoperative ISP levels in patients with pituitary adenomas and to determine whether elevated ISP is associated with tumor invasiveness, tumor volume, and pituitary apoplexy. Furthermore, by analyzing both pre- and postoperative endocrine profiles, we aimed to clarify whether ISP contributes to the development of hypopituitarism in these patients.

## Methods

### Patients

This single-center, consecutive, retrospective, observational study included 84 patients newly diagnosed with pituitary adenomas between January 2024 and March 2025 at the Department of Neurosurgery, First Affiliated Hospital of Bengbu Medical University. Patients with recurrent adenomas who had undergone previous surgery were excluded, as destruction of sellar structures in such cases could compromise the accuracy of intraoperative ISP measurements.This study was approved by the Ethics Committee of the First Affiliated Hospital of Bengbu Medical University. All participants provided informed consent to participate in this study, which was conducted in accordance with the Declaration of Helsinki.

All patients were conscious and had normal limb movement at admission. Common symptoms included varying degrees of headache, blurred vision, and polyuria. Some patients also presented with underlying comorbidities such as hypertension, diabetes mellitus, and hyperlipidemia. Among them, patients with pituitary adenoma apoplexy exhibited sudden worsening of headache and marked deterioration of vision. All patients underwent endoscopic transnasal transsphenoidal pituitary adenoma resection, during which ISP was measured intraoperatively. Hormone levels were assessed preoperatively and at 12 weeks postoperatively during follow-up.

### Clinical and hormonal evaluation

Hypopituitarism involves partial or complete loss of regulatory function in the following hypothalamic–pituitary axes: adrenocorticotropic hormone (ACTH), thyroid-stimulating hormone (TSH), gonadotropins, growth hormone (GH), and prolactin (PRL) axes (14). Patients may present with symptoms such as fatigue, cold intolerance, loss of appetite, amenorrhea, decreased libido, and infertility. All patients underwent endocrine hormone assessments preoperatively and at 12 weeks postoperatively, including measurements of serum prolactin, growth hormone, cortisol at 8:00 a.m., cortisol at 4:00 p.m., thyroid-stimulating hormone, follicle-stimulating hormone, and adrenocorticotropic hormone. When baseline hormone levels were insufficient for diagnosis, dynamic functional tests—such as the ACTH stimulation test—were performed to diagnose primary adrenal insufficiency. According to the reference ranges for normal hormone secretion, hyperprolactinemia was defined as a serum prolactin level **>**25 μg/L (excluding elevations caused by physiological factors, medications, or other conditions). Adrenocortical insufficiency and hypothyroidism were diagnosed comprehensively based on corresponding hormone levels, clinical symptoms, and imaging findings.

### Radiological evaluation

All patients underwent contrast-enhanced 3.0T pituitary MRI with gadopentetate dimeglumine prior to surgery. Tumor dimensions included height (H), width (W), and anteroposterior length (L). Maximum tumor width was measured on coronal images, while height and anteroposterior length were measured on sagittal images.

Tumor volume was calculated using 3D-Slicer software. Tumor margins were manually delineated on each visible slice, and automated interpolation was used to reconstruct a three-dimensional model. Tumor volume was then generated automatically. All measurements were performed independently by an experienced radiologist and a neurosurgeon, with final values calculated as the mean of both observers.

Sellar destruction was evaluated using the Hardy-Wilson classification: grade 0, encapsulated adenoma confined within the sellar diaphragm; grade I, normal-sized sella or minor expansion with tumor <10 mm; grade II, tumor ≥10 mm with enlarged but intact sella floor; grade III, localized erosion or destruction of the sella floor; and grade IV, extensive destruction of the sella base resulting in a “phantom sella,” with nearly invisible margins (15).

Cavernous sinus invasion was assessed using the Knosp classification on coronal contrast-enhanced T1-weighted MRI: grade 0, adenoma does not exceed the medial tangent of the internal carotid artery (ICA); grade 1, extension up to the intercarotid line; grade 2, extension between the intercarotid and lateral tangent lines; grade 3, extension beyond the lateral tangent of the ICA; and grade 4, complete ICA encasement with extension into all cavernous sinus compartments or invasion of the parasellar or middle cranial fossa (16).

**Figure 1:**
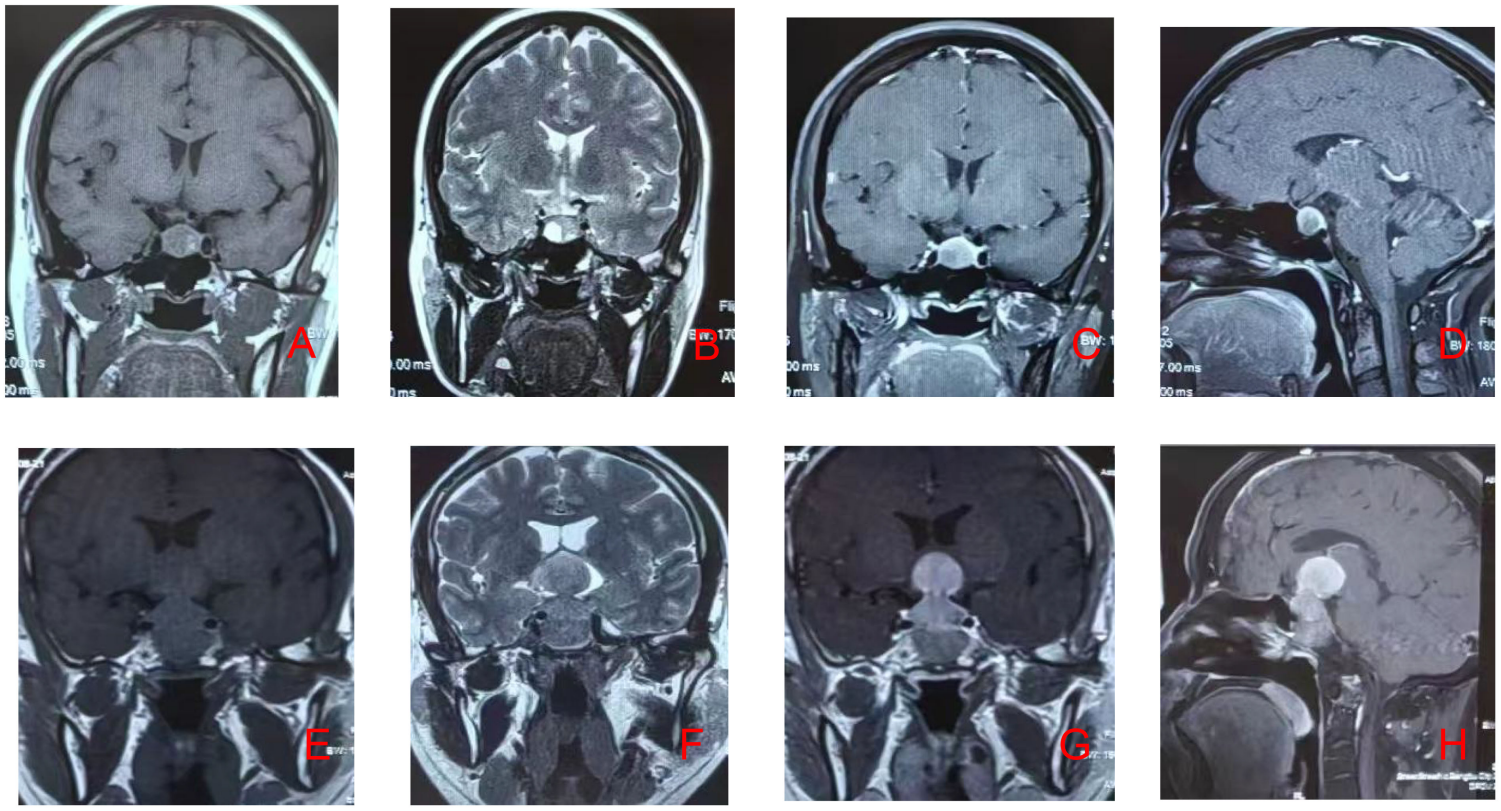
Preoperative MRI images of non-invasive and invasive pituitary adenomas. Panels **(A–D)** show MRI scans of a patient with a non-invasive pituitary adenoma, while panels **(E–H)** display MRI scans of another patient with an invasive pituitary adenoma.

Hardy-Wilson grades III–IV and Knosp grades 3–4 were considered invasive pituitary adenomas. Representative MRI images are shown in **Figure 1**.

### Measurement of ISP

All surgical procedures were performed using an endoscopic endonasal transsphenoidal approach. Surgeries were conducted by the same two neurosurgeons under standard general anesthesia with orotracheal intubation. Before the initiation of tumor resection, ISP was measured using an intracranial pressure monitoring device (Codman microsensor; manufactured at 11 Cabot Blvd., Mansfield, Massachusetts, 02048, USA). This device has been proven to provide accurate ISP measurements and is considered the standard clinical method for intracranial pressure monitoring (17).

**Figure 2:**
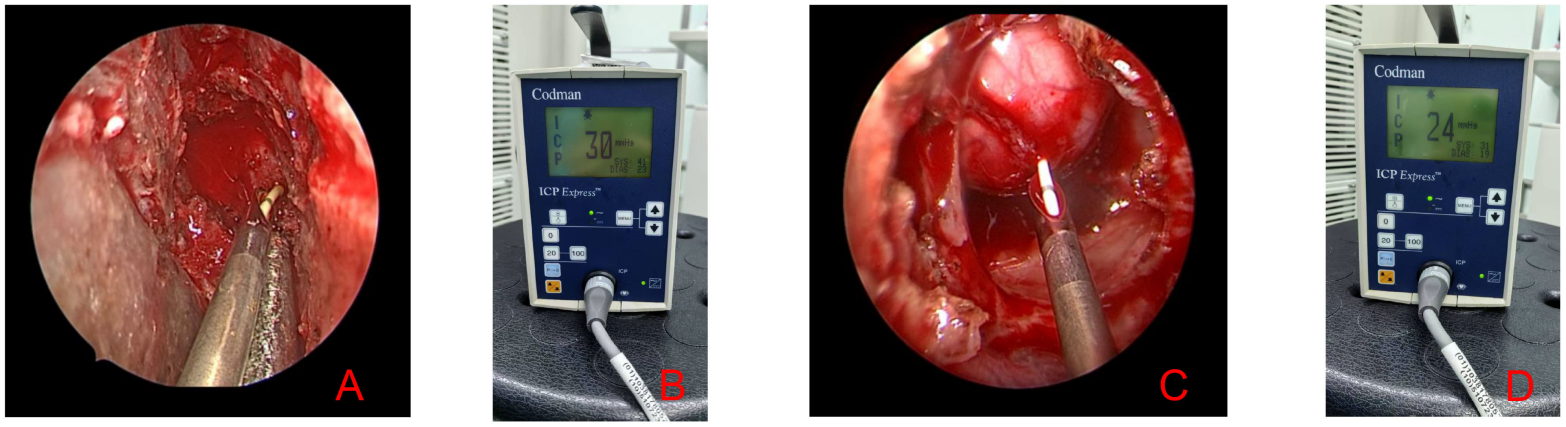
Intraoperative monitoring of intrasellar pressure (ISP). Panels **(A, C)** show the method of ISP measurement under direct endoscopic visualization, while panels **(B, D)** display the ISP values after stabilization of pressure waveforms.

The procedure was as follows: patients were placed in a supine position with the head tilted back 15°. Under endoscopic visualization, a small bony window (<2 mm in diameter) was drilled in the sellar wall. A dural incision of approximately 1.5 mm was then made to access the sellar cavity, taking care to avoid cerebrospinal fluid leakage. After calibration, the microsensor was inserted approximately 5 mm into the tumor. ISP values were recorded once pressure fluctuations stabilized. The intraoperative procedure is illustrated in **Figure 2**.

#### Histological evaluation

All tumor specimens underwent pathological examination. Immunohistochemical analysis was performed in conjunction with clinical presentation, imaging, and laboratory data to determine tumor subtype. Of the 84 patients, 9 had prolactin-secreting adenomas, 9 had growth hormone–secreting adenomas, 5 had adrenocorticotropic hormone (ACTH) adenomas, and 61 had non-functioning adenomas. No cases of TSH-secreting adenomas were identified.

Patients with prolactin-secreting adenomas were included in the ISP measurement dataset but excluded from correlation analyses between ISP and serum prolactin levels.

### Statistical analysis

The organized data were analyzed using SPSS 29.0 statistical software (SPSS IBM, Armonk, New York). ISP levels and tumor size were expressed as mean ± standard deviation (x̄ ± s). A t-test was used to analyze differences in ISP levels between groups with two variables, while one-way analysis of variance (ANOVA) was performed to compare ISP levels among groups with more than two variables. Categorical variables were presented as counts or percentages (%), and comparisons between groups were conducted using the chi-square test. Spearman correlation analysis was used to investigate the relationship between tumor volume parameters and intrasellar pressure. A P value < 0.05 was considered statistically significant.

## Results

### Patients’ clinical characteristics

Among the 84 patients included in this study, 42 were male and 42 female, with a mean age of 51.48 ± 12.62 years. Histological subtypes included 9 prolactin-secreting adenomas (10.71%), 9 growth hormone–secreting adenomas (10.71%), 5 ACTH adenomas (4.76%), and 61 non-functioning adenomas (72.62%). The mean tumor volume was 7.48 ± 3.27 cm³. There were 7 microadenomas (R < 10 mm), 70 macroadenomas (10 mm < R ≤ 40 mm), and 7 giant adenomas (R > 40 mm).

Clinical manifestations included headache, dizziness, visual impairment, and polyuria. Some patients had comorbid conditions such as hypertension, diabetes mellitus, or hyperlipidemia. Detailed clinical characteristics are summarized in **Table 1**.

**Table 1:** General clinical characteristics of 84 patients (omitted here, values consistent with original).

Category	N	%	ISP (mmHg)	χ²/t-value	*P*-value
Male	42	50	30.11 ± 6.95	1.05	0.30
Female	42	50	31.72 ± 7.10		
Age (years)	51.48 ± 12.62	/	/	/	/
PRL adenoma	9	10.71	29.81 ± 4.97	0.52	0.67
GH adenoma	9	10.71	28.54 ± 9.30		
ACTH adenoma	5	4.76	30.90 ± 5.32		
Nonfunctioning adenoma	61	72.62	31.43 ± 7.09		
Microadenoma	7	8.33	23.83 ± 4.39	4.70	0.01
Macroadenoma	70	83.33	31.16 ± 6.58		
Giant adenoma	7	8.33	33.74 ± 8.45		
Non-invasive (Knosp grade 0–2)	49	58.33	30.27 ± 6.75	3.11	<0.01
Invasive (Knosp grade 3–4)	35	41.67	35.06 ± 7.27		
Headache	42	50	30.61 ± 6.94	0.22	0.80
Visual impairment	32	38.10	31.20 ± 6.39		
Polyuria	4	4.76	29.03 ± 3.66		
Hypertension	24	28.57	32.35 ± 7.14	0.10	0.91
Diabetes mellitus	9	10.71	31.21 ± 8.14		
Hyperlipidemia	33	39.29	31.71 ± 7.22		

### ISP and tumor volume, tumor invasiveness

**Figure 3:**
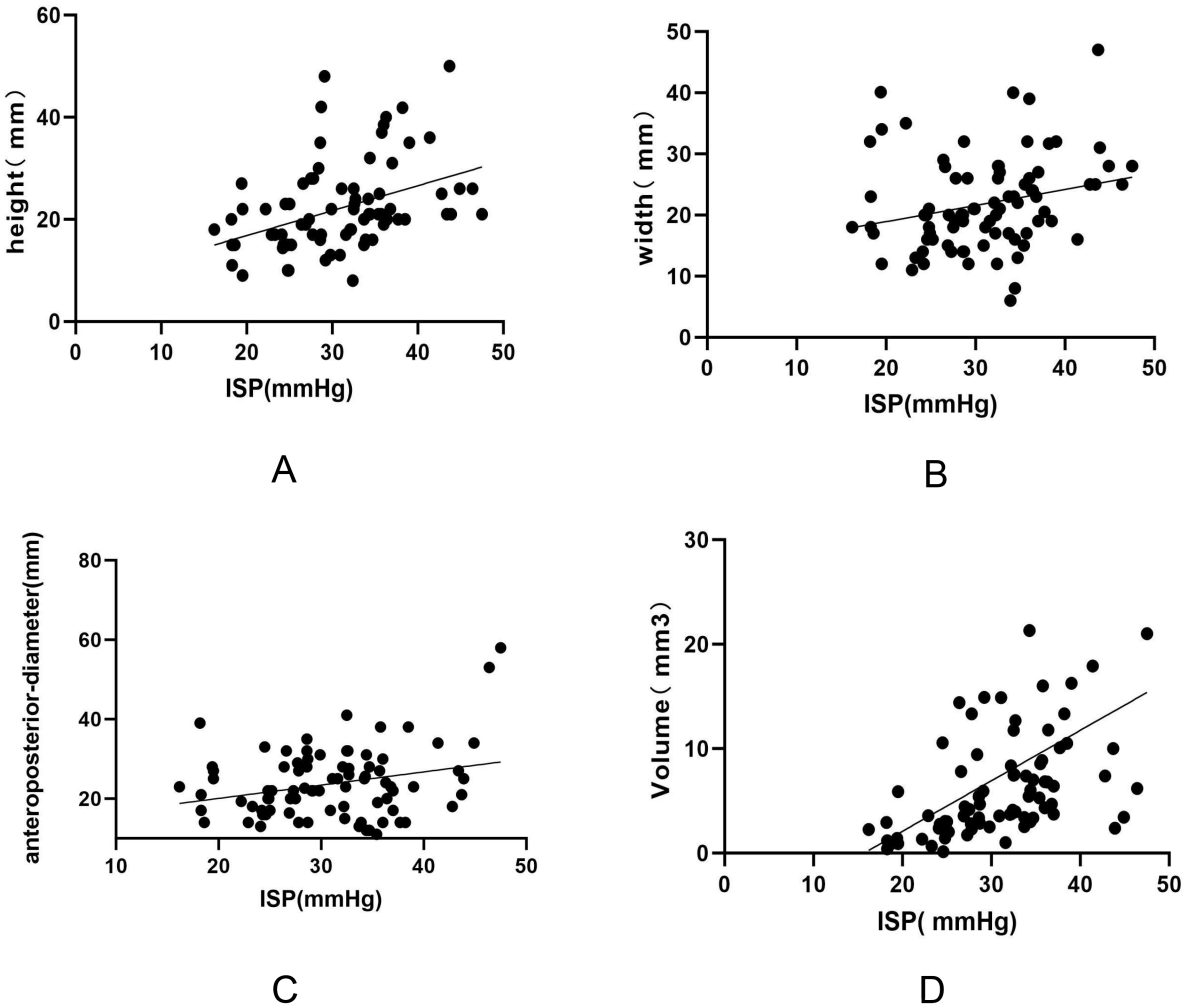
Correlation between ISP and pituitary adenoma size/volume (Knosp classification). Panel **(A)** shows the correlation between ISP and tumor height in the coronal plane; Panel **(B)** shows the correlation between ISP and tumor width in the sagittal plane; Panel **(C)** shows the correlation between ISP and tumor anteroposterior diameter; and Panel **(D)** shows the correlation between ISP and tumor volume.

To investigate the association between ISP and tumor invasiveness, Hardy-Wilson grading classified 45 patients as grade 0–II and 39 patients as grade III–IV. Knosp classification identified 49 patients as grade 0–2 and 35 patients as grade 3–4.The mean intraoperative ISP of all patients was 30.91 ± 7.03 mmHg. Patients with invasive pituitary adenomas, as defined by either Hardy-Wilson grade III–IV or Knosp grade 3–4, exhibited significantly higher ISP levels compared with non-invasive tumors (**Table 2**). In addition, ISP increased with tumor volume (**Table 3**), and ISP was positively correlated with tumor size (R² = 0.14, *p* < 0.01). Among the three dimensional parameters, tumor height demonstrated the strongest correlation with ISP (R² = 0.16, *p* < 0.01), followed by tumor width (R² = 0.06, *p* = 0.03) and anteroposterior diameter (R² = 0.07, *p* = 0.01). Multivariate analysis confirmed that tumor height exerted the strongest effect on ISP (*p* < 0.01) (**Figure 3**).

**Table 2:** Comparison of general characteristics between invasive and non-invasive pituitary adenomas.

Category	Total	Hardy-Wilson grade		*P*-value	Knosp grade		*P*-value
		0-II	III-IV		0-2	3-4	
N	84	45	39	/	49	35	/
Male (n)	42	23	19	0.83	22	20	0.27
Female (n)	42	22	20		27	15	
Age (years)	51.48 ± 12.62	48.78 ± 13.26	54.59 ± 11.21	0.34	48.67 ± 12.69	55.40 ± 11.58	0.74
Pituitary stalk compression	66	20	46	<0.01	24	42	<0.01
Pituitary stalk intact	18	12	6		13	5	
Intrasellar growth	45	38	7	<0.01	34	11	<0.01
Suprasellar growth	39	5	34		13	26	
Lateral growth	27	15	12	0.48	11	16	0.51
Symmetric growth	57	27	30		19	38	
ISP (mmHg), Mean ± SD	30.91 ± 7.03	29.13 ± 6.68	32.67 ± 6.75	0.02	30.27 ± 6.75	35.06 ± 7.27	<0.01

Hardy-Wilson grades 0–II represent non-invasive tumors, grades III–IV represent invasive tumors. Knosp grades 0–2 represent non-invasive tumors, grades 3–4 represent invasive tumors.

**Table 3:** Relationship between ISP, tumor invasiveness, and tumor size.

Classification	Tumor size	Invasive (Mean ± SD)	Non-invasive (Mean ± SD)	*T*-value	*P*-value
Knosp grade	Height (mm)	25.61 ± 9.63	19.39 ± 7.06	4.30	0.04
	Width (mm)	25.72 ± 9.09	19.43 ± 6.59	2.57	0.11
	Anteroposterior diameter (mm)	28.62 ± 9.43	20.18 ± 6.40	2.20	0.14
	Volume (cm²)	11.52 ± 6.53	4.59 ± 2.13	6.94	<0.01
	ISP (mmHg)	35.06 ± 7.27	30.27 ± 6.75	3.11	<0.01
Hardy-Wilson grade	Height (mm)	25.76 ± 9.40	18.71 ± 6.65	5.63	0.02
	Width (mm)	25.42 ± 8.94	19.13 ± 6.46	3.54	0.06
	Anteroposterior diameter (mm)	27.27 ± 9.00	20.60 ± 7.44	0.07	0.79
	Volume (cm²)	10.99 ± 5.94	4.43 ± 2.07	6.94	<0.01
	ISP (mmHg)	32.67 ± 6.75	29.13 ± 6.68	2.41	0.02

#### ISP and hypopituitarism

Endocrine function was assessed preoperatively and at 12 weeks postoperatively in all 84 patients. Elevated ISP was significantly associated with a higher preoperative incidence of adrenal insufficiency (*p* = 0.02), hypothyroidism (*p* = 0.04), and hyperprolactinemia (*p* = 0.03). However, at 12 weeks after surgery, ISP showed no significant correlation with postoperative adrenal insufficiency (*p* = 0.15), hypothyroidism (*p* = 0.80), or hyperprolactinemia (*p* = 0.15) (**Table 4**).

**Table 4:** Relationship between ISP and endocrine dysfunction preoperatively and 12 weeks postoperatively.

Category	Endocrine function	N	ISP (mmHg)	*T*-value	*P*-value
Preoperative	Normal	11	28.03 ± 5.83	/	/
	Cortisol insufficiency	22	33.41 ± 6.23	2.37	0.02
	Hypothyroidism	11	33.75 ± 6.40	2.19	0.04
	Hyperprolactinemia	40	32.65 ± 6.23	2.21	0.03
Postoperative (12 weeks)	Normal	68	30.56 ± 6.25	/	/
	Cortisol insufficiency	5	31.38 ± 7.94	2.23	0.15
	Hypothyroidism	3	29.40 ± 8.73	0.29	0.80
	Hyperprolactinemia	8	31.39 ± 3.92	2.32	0.15

### ISP and pituitary apoplexy

Among the 84 patients, 18 were diagnosed with pituitary apoplexy and 66 without. Comparative analysis demonstrated no significant association between ISP and pituitary apoplexy. Instead, hypertension was identified as a strong independent risk factor for apoplexy (*p* < 0.01). Additionally, adrenal insufficiency and hypothyroidism were significantly associated with hemorrhagic apoplexy (*p* < 0.01 for both).

No significant differences were observed between apoplexy and non-apoplexy groups with respect to age, sex, tumor volume, or tumor invasiveness (**Table 5**).

**Table 5:** Relationship between ISP and pituitary apoplexy.

Category	Pituitary apoplexy (n=18)	Non-apoplexy (n=66)	χ²/F-value	*P*-value
N	18	66	/	/
Age (years)	45.50 ± 10.64	53.11 ± 12.69	0.42	0.52
Sex (male/female)	12/6	30/36	2.55	0.11
Tumor volume (cm³)	11.38 ± 11.93	6.41 ± 8.20	1.77	0.19
Hypertension	13	11	21.39	<0.01
Cortisol insufficiency	10	12	7.59	<0.01
Hypothyroidism	6	5	8.25	<0.01
Hyperprolactinemia	12	28	3.33	0.07
Invasive (Knosp grade 3–4)	7	28	0.07	0.79
Non-invasive (Knosp grade 0–2)	11	38		
ISP (mmHg)	30.33 ± 7.46	31.07 ± 6.96	1.77	0.22

## Discussion

In this study, we systematically analyzed clinical data from 84 patients with pituitary adenomas to explore the relationship between intrasellar pressure (ISP) and tumor invasiveness, tumor volume, hypopituitarism, and pituitary apoplexy. The principal findings were: (1) ISP was significantly higher in invasive pituitary adenomas compared with non-invasive tumors; (2) ISP increased with tumor volume, with tumor height showing the strongest correlation; (3) elevated ISP was associated with a higher preoperative incidence of adrenal insufficiency, hypothyroidism, and hyperprolactinemia, but not with postoperative hypopituitarism at 12 weeks; and (4) ISP was not significantly correlated with pituitary apoplexy.

### ISP and tumor invasiveness

ISP reflects the pressure within the sellar cavity and represents the physiological and pathological state of the pituitary and its surrounding structures (18). Pituitary adenomas often extend laterally into the cavernous sinus or superiorly into the suprasellar region, and tumor invasiveness is typically classified by the Hardy-Wilson and Knosp systems (6). Our findings demonstrated that ISP was significantly elevated in invasive tumors.

Several mechanisms may account for this association. First, mechanical compression: tumor enlargement increases ISP, which in turn promotes growth along the path of least resistance, such as through the diaphragma sellae or weakened bony margins (19). Second, ischemia and hypoxia: higher ISP can impair local perfusion, inducing hypoxia and activating hypoxia-inducible factor-1α (HIF-1α) signaling, thereby stimulating matrix metalloproteinase (MMP) activity and invasive behavior (20). Third, inflammation and fibrosis: ISP elevation may trigger inflammatory responses and dural thickening, yet adenomas can overcome these barriers through proteolytic enzymes such as MMP-9 (21). Collectively, these mechanisms suggest that ISP not only reflects tumor invasiveness but may also contribute to its progression, although further mechanistic validation is warranted.

### ISP, tumor volume, and correlation with tumor height

MRI remains the gold standard for the diagnosis and monitoring of pituitary adenomas (22). Previous studies have suggested that increasing tumor volume leads to expansion of intrasellar contents, resulting in elevated ISP (11). Our data confirm these observations and further demonstrate that tumor height is the most important dimensional factor associated with ISP.

The sella turcica is a rigid cavity with limited compliance (normally 1–2 mL) (23). As adenomas enlarge beyond this compliance threshold, ISP rises sharply (5, 24). Typical MRI signs, such as the “snowman” or “hourglass” configuration, are often indicative of higher ISP (25). Interestingly, although cavernous sinus invasion (Knosp grades 3–4) may transiently alleviate pressure through lateral extension, further tumor growth ultimately re-elevates ISP (26).

### ISP and hypopituitarism

Hypopituitarism, or pituitary insufficiency, is a clinical syndrome caused by inadequate secretion of one or more anterior pituitary hormones, with or without involvement of the posterior pituitary. The primary mechanism underlying its development is that a slowly growing pituitary adenoma obstructs the pituitary portal blood flow. In addition, tumor expansion within the rigid confines of the sella turcica leads to an increase in intrasellar pressure (ISP). Elevated ISP compresses the pituitary stalk, and together these factors interfere with the delivery of hypothalamic hormones—such as TRH, CRH, and GnRH—resulting in secondary hypothyroidism, adrenal insufficiency, or hypogonadism (27, 28). Prolactin is synthesized and secreted by lactotroph cells in the anterior pituitary, and the core mechanism of hyperprolactinemia is a disruption of the hypothalamic–pituitary regulatory control of prolactin secretion. Dopamine is the major inhibitory factor regulating prolactin release; it binds to D2 receptors on lactotrophs and strongly suppresses prolactin synthesis and secretion. Therefore, any factor that interferes with dopamine synthesis, release, or transport will remove this inhibitory effect and lead to elevated prolactin levels.When pituitary adenomas enlarge and extend suprasellarly, the consequent elevation of ISP externally compresses, stretches, or directly invades the pituitary stalk. Such compression may result in edema, deformation, or structural damage of the stalk, and the pituitary portal vessels within it may become compressed or obstructed (29). As a result, hypothalamic dopamine cannot be effectively delivered to lactotrophs in the anterior pituitary. With the loss of dopaminergic inhibition, lactotroph cells begin to secrete prolactin autonomously, ultimately leading to the development of hyperprolactinemia (30). Consistent with these mechanisms, we observed significant associations between elevated ISP and preoperative adrenal insufficiency, hypothyroidism, and hyperprolactinemia. However, no such correlation was found at 12 weeks postoperatively, which may reflect preexisting irreversible pituitary damage, resection of residual normal tissue, or intraoperative stalk injury (31).

### ISP and pituitary apoplexy

Pituitary apoplexy is a potentially life-threatening condition characterized by ischemia or hemorrhage within the adenoma. In our cohort, ISP was not significantly associated with apoplexy. Instead, hypertension emerged as an independent risk factor, while adrenal insufficiency and hypothyroidism were linked to hemorrhagic apoplexy.

Prior reports suggested that large adenomas with markedly elevated ISP may predispose to vascular rupture due to fragile neovascularization (32). MRI features such as the “snowman sign” have also been correlated with apoplexy (25), and elevated ISP may impair microcirculation in the portal system, leading to ischemic necrosis and hemorrhage (33). The discrepancy between these reports and our findings may be attributed to the limited number of apoplexy cases in our cohort (18/84 patients). Given that apoplexy occurs in only 2–12% of adenoma cases (34), larger prospective studies are needed to clarify this relationship.

To our knowledge, this is the first study to comprehensively examine the association between intraoperative ISP and multiple clinical outcomes, including tumor invasiveness, volume, endocrine dysfunction, and apoplexy. Our findings provide novel evidence that ISP may serve as both a marker and a potential mediator of pituitary adenoma behavior. Clinically, intraoperative ISP measurement may aid in prognostic stratification and postoperative endocrine management.

## Conclusion

In summary, our study demonstrates that elevated intraoperative ISP is closely associated with preoperative endocrine dysfunction in patients with pituitary adenomas and serves as an important indicator of tumor invasiveness. However, no correlation was observed between intrasellar pressure and postoperative hypopituitarism or pituitary apoplexy.

## Limitations

Although this study demonstrates the relationship between ISP and tumor volume, tumor invasiveness, preoperative hypopituitarism, hyperprolactinemia, as well as postoperative endocrine outcomes—an issue of high clinical relevance—it has several limitations. These include a relatively small sample size, its retrospective design, and a short follow-up duration, which may introduce potential bias. Future studies with larger cohorts and longer follow-up periods are needed to provide more robust and in-depth evidence.

## Data Availability

The original contributions presented in the study are included in the article/supplementary material, further inquiries can be directed to the corresponding author/s.

## References

[1] Ho KKY Melmed S . Pituitary adenomas: biology, nomenclature and clinical classification. Rev Endocr Metab Disord. (2025) 26:137–46. doi: 10.1007/s11154-025-09944-x, PMID: 39862335

[2] Voelker RA . What are pituitary adenomas? JAMA. (2023) 330:2224. doi: 10.1001/jama.2023.15248, PMID: 37948091

[3] Daly AF Beckers A . The epidemiology of pituitary adenomas. Endocrinol Metab Clin North Am. (2020) 49:347–55. doi: 10.1016/j.ecl.2020.04.002, PMID: 32741475

[4] Fleseriu M Christ-Crain M Langlois F Gadelha M Melmed S . Hypopituitarism. Lancet. (2024) 403:2632–48. doi: 10.1016/S0140-6736(24)00342-8, PMID: 38735295

[5] Lefevre E Chasseloup F Hage M Chanson P Buchfelder M Kamenický P . Clinical and therapeutic implications of cavernous sinus invasion in pituitary adenomas. Endocrine. (2024) 85:1058–65. doi: 10.1007/s12020-024-03877-2, PMID: 38761347

[6] Bello-Lemus VH Torres-Zapiain F Murillo-Ortiz BO . Patrón de crecimiento invasor en adenomas hipofisarios con inmunohistoquímica hormonal positiva [Pattern of invasive growth of pituitary adenomas with positive hormonal immunohistochemistry. Rev Med Inst Mex Seguro Soc. (2025) 63:e6409. doi: 10.5281/zenodo.14617045, PMID: 40273459 PMC12043349

[7] Rouf S Berrabeh S Zarraa L Latrech H . Knosp and revised Knosp classifications predict non-functioning pituitary adenoma outcomes: a single tertiary center experience. J Med Life. (2024) 17:1007–11. doi: 10.25122/jml-2024-0015, PMID: 39781308 PMC11705471

[8] Flores-Rabasa R González-Almazán JA Cortés-Contreras AP Méndez-García LA Velasco F Navarro-Olvera JL . Pre- and post-clinical-radiological and surgical evaluation of patients with pituitary adenoma and metabolic syndrome. Int J Neurosci. (2024) 134:1003–12. doi: 10.1080/00207454.2023.2203836, PMID: 37060337

[9] Ito M Mitobe Y Hiraka T Kanoto M Sonoda Y . Changes in vascular supply pattern associated with growth of nonfunctioning pituitary adenomas. Surg Neurol Int. (2022) 13:481. doi: 10.25259/SNI_879_2022, PMID: 36324967 PMC9610044

[10] Simander G Eriksson PO Viirola S Lindvall P Koskinen LD . Complications following endoscopic transsphenoidal surgery for pituitary adenoma-special focus on intrasellar pressure. Acta Neurochir (Wien). (2025) 167:83. doi: 10.1007/s00701-025-06495-7, PMID: 40105980 PMC11923022

[11] Simander G Eriksson PO Lindvall P Koskinen LD . Intrasellar pressure in patients with pituitary adenoma - relation to tumour size and growth pattern. BMC Neurol. (2022) 22:82. doi: 10.1186/s12883-022-02601-9, PMID: 35264140 PMC8905730

[12] Simander G Dahlqvist P Oja L Eriksson PO Lindvall P Koskinen LD . Intrasellar pressure is related to endocrine disturbances in patients with pituitary tumors. World Neurosurg. (2023) 175:e344–51. doi: 10.1016/j.wneu.2023.03.085, PMID: 36966914

[13] Kajal S Ahmad YES Halawi A Gol MAK Ashley W . Pituitary apoplexy: a systematic review of non-gestational risk factors. Pituitary. (2024) 27:320–34. doi: 10.1007/s11102-024-01412-0, PMID: 38935252

[14] Akirov A Rudman Y Fleseriu M . Hypopituitarism and bone disease: pathophysiology, diagnosis and treatment outcomes. Pituitary. (2024) 27:778–88. doi: 10.1007/s11102-024-01391-2, PMID: 38709467

[15] Araujo-Castro M Acitores Cancela A Vior C Pascual-Corrales E Rodríguez Berrocal V . Radiological knosp, revised-knosp, and hardy-wilson classifications for the prediction of surgical outcomes in the endoscopic endonasal surgery of pituitary adenomas: study of 228 cases. Front Oncol. (2022) 11:807040. doi: 10.3389/fonc.2021.807040, PMID: 35127519 PMC8810816

[16] Giardini E Barbosa MA Ventura N da Mata Pereira PJ Guasti A Niemeyer P . Improving the radiological prediction of surgical resection of nonfunctioning pituitary adenomas. J Endocrinol Invest. (2025) 48:701–9. doi: 10.1007/s40618-024-02479-z, PMID: 39499435

[17] Zhang J Yin T Ding C Gu J Zhu B Li J . The "Double-peak" Pattern of pituitary adenoma intrasellar pressure and its effects on the microvascular structure. World Neurosurg. (2021) 151:e137–45. doi: 10.1016/j.wneu.2021.03.146, PMID: 33831613

[18] Kortbawi R Ray A Selman WR Arafah BM . Recovery of pituitary function in patients with apoplexy immediately after surgical resection of necrotic tumors. Endocrine. (2025) 89(1):222–31. doi: 10.1007/s12020-025-04222-x, PMID: 40227461

[19] Anderson C Akbar N Colley P . Reconstruction of skull base defects in pituitary surgery. Otolaryngol Clin North Am. (2022) 55:449–58. doi: 10.1016/j.otc.2022.01.004, PMID: 35365317

[20] Yu S Duan Q Niu C Mu C . Activation of the HIF1α/TIMP1/MT6-MMP pathway is associated with invasion in pituitary null cell adenomas. Endocr Relat Cancer. (2025) 32:e240146. doi: 10.1530/ERC-24-0146, PMID: 39670877

[21] Thé B Freire B AC Jallad RS Batista RL Glezer A Machado MC Ochman G . Expression of MMP-2, MMP-9 and TIMP-2 in pituitary tumors and their relationship with cavernous sinus invasion. Endocr Oncol. (2025) 5:e240037. doi: 10.1530/EO-24-0037, PMID: 39810846 PMC11728929

[22] Yuzkan S Erkan B Dogukan FM Ozkiziltan U Balsak S Arslan FZ . Distinguishing pituitary metastasis and pituitary neuroendocrine tumors through conventional MR imaging and clinical features. AJNR Am J Neuroradiol. (2024) 45:1063–9. doi: 10.3174/ajnr.A8302, PMID: 38871368 PMC11383409

[23] Ilahi S Ilahi TB . Anatomy, Adenohypophysis (Pars Anterior, Anterior Pituitary). In: StatPearls. StatPearls, Treasure Island (FL (2022)., PMID: 30085581

[24] Lin K Feng T Mu S Wang S . Interaction between pituitary adenoma growth and the diaphragma sellae. Asian J Surg. (2022) 45:1638–9. doi: 10.1016/j.asjsur.2022.03.066, PMID: 35367094

[25] Bukhari K Sharma V Gupta S Motazedi A . The snowman sign in a patient with pituitary tumor apoplexy. J Community Hosp Intern Med Perspect. (2021) 11:416–7. doi: 10.1080/20009666.2021.1898086, PMID: 34234919 PMC8118409

[26] Ali HM Leland EM Stickney E Lohse CM Iyoha E Valappil B . Multi-center study on sellar reconstruction after endoscopic transsphenoidal pituitary surgery. Int Forum Allergy Rhinol. (2024) 14:1558–67. doi: 10.1002/alr.23382, PMID: 38884280

[27] Gounden V Anastasopoulou C Jialal I . Hypopituitarism. In: StatPearls. StatPearls, Treasure Island (FL (2023). 29262053

[28] Hussein Z Marcus HJ Grieve J Dorward N Kosmin M Fersht N . Pituitary function at presentation and following therapy in patients with non-functional pituitary macroadenomas: a single centre retrospective cohort study. Endocrine. (2023) 82:143–51. doi: 10.1007/s12020-023-03434-3, PMID: 37389717 PMC10462492

[29] Shapiro M Sharashidze V Nossek E Sen C Rutledge C Chung C . Superior hypophyseal arteries: angiographic re-discovery, comprehensive assessment, and embryologic implications. J neurointerv Surg. (2024) 17:e41–6. doi: 10.1136/jnis-2023-020922, PMID: 37875341

[30] Mann JA Bereznicki C Lithgow K . Workup of hyperprolactinemia. CMAJ. (2025) 197(14):E390–1. doi: 10.1503/cmaj.241710, PMID: 40228839 PMC12007944

[31] Meij BP van Stee LL . Transsphenoidal surgery for pituitary tumors. Vet Clin North Am Small Anim Pract. (2025) 55:95–118. doi: 10.1016/j.cvsm.2024.07.009, PMID: 39227253

[32] Mayol Del Valle M De Jesus O . Pituitary Apoplexy. In: StatPearls. StatPearls Publishing, Treasure Island (FL (2023). 32965873

[33] Biagetti B Cordero Asanza E Pérez-López C Araujo-Castro M Camara R Guerrero-Pérez F . Pituitary Apoplexy: Comorbidities, Management, and Outcomes-A Spanish Observational Multicenter Study [published correction appears in J Clin Endocrinol Metab. J Clin Endocrinol Metab. (2025) 110:e1811–20. doi: 10.1210/clinem/dgae649. 2025 Jun 17;110(7):e2432. doi: 10.1210/clinem/dgaf222.]., PMID: 39298667

[34] Briet C Salenave S Bonneville JF Laws ER Chanson P . Pituitary apoplexy. Endocr Rev. (2015) 36:622–45. doi: 10.1210/er.2015-1042, PMID: 26414232

